# Understanding the Relationship Between State Forgiveness and Psychological Wellbeing: A Qualitative Study

**DOI:** 10.1007/s10943-016-0188-9

**Published:** 2016-03-01

**Authors:** Sadaf Akhtar, Alan Dolan, Jane Barlow

**Affiliations:** 1 Warwick Medical School, University of Warwick, Coventry, UK; 2 Centre for Lifelong Learning, University of Warwick, Coventry, UK

**Keywords:** Forgiveness, Psychological wellbeing, Religion, Spirituality, Interpersonal relationships

## Abstract

Over the last 20 years, increasing attention has been given to associations between dispositional forgiveness and specific mental health problems. However, few studies have assessed whether forgiving real-life interpersonal hurts may be related to diverse psychological health outcomes. The present study addresses this gap by investigating, in depth, relationships between perceptions about state forgiveness and a variety of mental wellbeing outcomes as well as exploring perceptions about the factors that may modify such effects. Developing an understanding of a forgiveness wellbeing relationship is of relevance to healthcare workers, researchers and policy makers with an interest in improving public health. In-depth semi-structured interviews were conducted, and data were analysed using grounded theory methods. From England and Ireland, eleven adults who were affiliated with religious/spiritual and secular/atheist groups were recruited using purposive and convenience sampling methods. Key themes that appeared to be related to the effects of unforgiveness were: increases in negative affect; reduction in cognitive abilities and barriers to psychological and social growth. For the majority of participants, state forgiveness had strong ties to participants perceived sense of mental wellbeing, including reductions in negative affect, feeling positive emotions, positive relations with others, spiritual growth, a sense of meaning and purpose in life as well as a greater sense of empowerment. The data also revealed a number of factors that may positively or negatively influence a forgiveness–wellbeing link such as: viewing an offender as spiritually similar or different, responsibility/karma, blaming, wanting restitution/apology as well as practices such as meditation and prayer. The findings suggest that forgiving a range of real-life interpersonal offences may be an important determinant of psychological wellbeing, particularly among religious/spiritual populations. Further research is, however, needed.

## Introduction

Interpersonal hurts are common, and they can occur due to experiences of physical or emotional abuse, or in other cases, due to conflicts relating to feeling unacknowledged or rejected (Krug et al. 2002; Diblasio 1998). Consequently, hurt people may develop chronic emotions of anger or hostility, which can result in the development of physical and mental health problems (Goldman and Wade 2012). One method of coping with such hurt and its negative consequences is forgiveness, which entails letting go of recurrent anger, hostility or the desire for revenge and choosing to develop compassion, sympathy or pity for an offender (Enright and Fitzgibbons 2000; Wade and Worthington 2005). Because forgiveness aims to reduce negative thoughts, emotions and behaviours, resulting from interpersonal hurts, researchers, clinicians and policy makers with an interest in promoting population wellbeing are increasingly interested in the health benefits of this practice (Macaskill 2005).

## Forgiveness and Mental Wellbeing Outcomes: Empirical Evidence

A number of experimental studies have been conducted to assess whether forgiveness therapy reduces common mental health problems and, in a few cases, whether it promotes marital satisfaction, gratitude, positive affect, self-esteem, hope and spiritual health (Lundahl et al. 2008). Meta-analyses assessing the effectiveness of these forgiveness interventions have found that they reduce depression, anxiety and stress and increase subjective wellbeing (Baskin and Enright 2004; Lundahl et al. 2008; Wade et al. 2013). However, prior experimental research largely assesses the effects of group based manualised forgiveness interventions, and few of these studies examine whether forgiveness promotes general wellbeing outcomes.

A number of correlational studies have also been conducted and lend some support to the findings of prior experimental research. A literature review (Toussaint and Webb 2005) found that the majority of studies using correlational survey designs detected associations between trait forgiveness, which assessed participants propensity to forgive in a given situation, and depression and anxiety. However, only two studies found associations between state (real life) forgiveness and mental health. Moreover, even fewer studies had investigated whether such forgiveness is associated with reductions to a wider range of mental health problems.

In addition, a few correlational studies have examined relationships between dispositional (i.e. trait) forms of forgiveness and subjective wellbeing. Some have found only a weak association between trait forgiveness and a single measure of life satisfaction (Sastre et al. 2003), whilst others have detected positive associations between dispositional forgiveness and life satisfaction (Krause and Ellison 2003; Allemand et al. 2012; Chan 2013). One prior study examined the effects of trait forgiveness on eudaimonic wellbeing and found an association with improved relationships with others (Hill and Allemand 2010). Only two studies have investigated links between state forgiveness and subjective wellbeing. One of these studies found reductions in (state) unforgiveness to be strongly correlated with positive affect (Toussaint and Friedman 2009). The second study found that state forgiveness (of another person) was associated with increased happiness, although to a small degree (Maltby et al. 2005). Thus, the majority of correlational research either assesses associations between dispositional forgiveness and depression or anxiety, or in a few cases, links with subjective levels of happiness.

No qualitative studies have explored the effects of forgiving real-life hurts. However, one study investigating forgiveness in the lives of religious people found that participants felt that they experienced an increase in positive emotions (e.g. peace, joy, calmness, contentment and gratitude) as well as improved relations with the wrongdoer as a result of practicing forgiveness (Kidwell et al. 2012).

Therefore, the majority of prior research explores the effects of group-based forgiveness therapy or dispositional (trait) forgiveness on specific mental health problems, or in a few cases, connections with subjective levels of happiness. There is as such limited research exploring in more detail the perceptions of individuals who have practiced forgiveness (state) in response to a real-life interpersonal hurt in order to better understand whether forgiveness is linked to wider domains of functioning. At present, very little is also known about factors facilitating or obstructing forgiveness among secular and diverse religious samples. Thus, a further aim of this study is to examine whether specific religious or secular beliefs and practices are perceived to be associated with changes in forgiveness and general wellbeing, prior to the conduct of further research.

## Aims of this Study

The aims of this qualitative study were to: (1) explore the perceived effects of practicing state forgiveness on a variety of mental wellbeing outcomes and (2) to explore factors influencing a forgiveness–wellbeing relationship.

## Methods

Eleven English-speaking adults affiliated with New Religious, Buddhist, Muslim and Secular/Atheist groups were recruited using purposive and snowball sampling methods through a variety of means such as social media (Facebook groups), direct email and telephone. Participants that met the criteria of having practiced forgiveness in response to an interpersonal hurt were invited to take part in the study. The types of hurts experienced by participants related to parental love deprivation, hurt by romantic partners and feelings of neglect within the context of work relationships. Six respondents were recruited from England and three were recruited from Ireland. Most were male (8 males and 3 females), ranging in age from 27 to 50 years. The average age of respondents was 36 years. Eight participants were white, two were Pakistani and one was black. Four participants had completed a higher degree, five had completed college-level qualifications and two had completed secondary education. The sample included participants affiliated with the following religious groups: A Course in Miracles, ACIM (*N* = 5), Muslim (*N* = 2), Buddhist (*N* = 1), Theosophist (*N* = 2) and secular/atheist (*N* = 1). Table 1 shows the profile of the interviewed participants.

**Table 1:** Demographic characteristics

Respondent	Gender	Ethnicity	Education	Age	Affiliation
				Mean = 36	
Brandon	Male	White	College	26–35	A Course in Miracles
Quinn	Male	White	College	36–45	A Course in Miracles
Bill	Male	White	College	46–55	A Course in Miracles
Alan	Male	White	College	26–35	Theosophy
Jax	Male	White	College	26–35	Buddhist
Trisha	Female	White	University	46–55	A Course in Miracles
Amira	Female	Asian	School	36–45	Muslim
Rana	Female	Asian	School	36–45	Muslim
Stuart	Male	White	University	46–55	A Course in Miracles
Lyndon	Male	Black	University	36–45	Theosophy
Alfred	Male	White	University	26–35	Secular/Atheist

### Procedure

To recruit participants, religious group organisers and members (i.e. A Course In Miracles, Buddhist, Muslim, Theosophist and Secular/Atheists) were contacted via telephone, the Internet (Facebook) and directly via email. Group organisers were also asked to assist in recruiting affiliate members and subsequently emailed information about the study to their members. All participants were provided with detailed information sheets about the research before consenting to take part in the study. Where appropriate, participants were also sent a list of questions they would be asked during the interviews. Arrangements were then made for the respondents to be interviewed. All participants were made aware that they could withdraw from the study at any point, without giving a reason.

In-depth semi-structured interviews were conducted. Some participants (*N* = 4) were based within the Midlands and surrounding areas, and so it was possible to travel to them. However, where distance and recruiting were an issue, interviews (*N* = 7) were carried out via video link (i.e. Skype). All participants were informed that interviews would take approximately 1–2 h; the majority of interviews lasted for approximately 80 min. A semi-structured interview schedule was used to elicit responses from participants regarding the process of forgiveness and its effects. All questions were open-ended, and, with permission, all interviews were audio-recorded and transcribed.

Grounded theory (GT) methods were applied to analyse qualitative data. These procedures consisted of the following steps: (1) repeated reading of the transcript; (2) writing memos regarding what may be emerging from the data; (3) open coding involving the separation of data to define concepts such as thoughts, ideas and meanings contained within blocks of raw data; (4) constant comparison such as assessing similarities and differences in emerging concepts within each transcript and across participants; (5) classifications where similar and different concepts were grouped together; (6) categorisation where concepts were developed into categories which were further defined in relation to their properties and dimensions; and (7) axial or intermediate coding (which often occurred concurrently alongside open coding). This final stage of coding concerned relating concepts/categories to each other, such as assessing relationships between themes.

### Ethics

This research was reviewed and given favourable opinion by the Biomedical and Scientific Research Ethics Committee at the University of Warwick.

## Results

### Perceived Effects of Unforgiveness

All of the participants spoke about the negative consequences that a lack of forgiveness had on their mental health and wellbeing. **A**nalysis of participant’s accounts revealed three key themes that appeared to be related to the effects of unforgiveness. These were: negative effect on mental health such as how participants felt; negative effect on participant’s mental health such as participant’s cognitive abilities; and barriers to growth that were both psychological and social. There appeared to be no obvious distinctions in these themes on the basis of religious/spiritual affiliation.

### Negative Effect on Mental Health/Affective

All participants described a variety of affective states resulting from unforgiveness, which on the whole, appeared to prevent them from adequately functioning emotionally. One Theosophist participant illustrated this theme by stating the following:

Fuming, you know when you’re in a state of bitterness, when you can’t really see or reflect properly but you’re just completely bitter and emotionally disturbed. (Lyndon, Theosophist)

Many said that they felt depressed (Alan, Theosophist), stressed and ‘no sense of peace’ (Bill, ACIM). Some also described feeling fear (Brandon, ACIM), guilt (Trisha, ACIM) and a lack of confidence (Rana, Muslim), whilst other examples included feeling a ‘lack of energy’ (Jax, Buddhist), ‘static’ and generally ‘less dynamic’ (Lyndon, Theosophist).

Stuart (ACIM) described how negative emotions of unforgiveness made him feel that he could not change his state, whilst Amira (Muslim) indicated that a lack of forgiveness resulted in a strong physiological response whereby she experienced feeling an intense negative reaction towards the offender. Another participant (Quinn, ACIM) said that he was unable to sleep, experienced intense anger as well as mood disorder, and Trisha (ACIM) indicated that holding on to grievances and not forgiving caused unhappiness and a lot of fear.

### Negative Effect on Mental Health/Cognitive

In addition to highlighting how unforgiveness negatively affected the way that participants felt, they also indicated that it negatively affected aspects of their cognitive health such that their thought processes appeared to be overwhelmingly negative. For example, many appeared to experience an ‘inability to think clearly’ (Rana, Muslim), whilst others indicated the presence of suicidal thoughts and of ‘harming others’ (Quinn, ACIM). On the whole, and linked to the previous theme, participants appear to have experienced a state whereby they were unable to feel or think constructively.

### Barriers to Psychological Growth and Social Wellbeing

A recurring theme among participants was that unforgiveness is a barrier to growth whereby many felt they were unable to develop or move forward psychologically or in terms of their social wellbeing. For instance, participants spoke of how a lack of forgiveness ‘freezes your mind’ making you ‘less dynamic’ (Trisha, ACIM) as well as ‘emotionally and cognitively slow’ (Lyndon, Theosophist). One participant described how her vengeance and anger made her feel as though she could not move forward:

It stops you from getting on with life, if you can’t forgive you’re stuck in that rut, stuck there in that place. I feel that. I felt I moved on so much with my sister in law (and husband) from where we were but I can’t move on from that particular person (brother in law). (Amira, Muslim)

Others also commented on how it negatively affected wider dimensions of wellbeing such as general relationships because the anger and resentment they felt were preventing them from forming new relationships. This was illustrated in a statement made by the secular/atheist respondent:

I do remember being quite bitter, which didn’t help with the ability to form relationships with anyone else. (Alfred, secular/atheist)

Overall, participant’s accounts suggested that unforgiveness did not just negatively effect how they felt; it also appeared to have a detrimental effect on their cognitive health and social wellbeing.

### Perceived Effects of Forgiveness

For the majority of participants, state forgiveness had strong ties to their perceived sense of mental wellbeing, including reductions in negative affect, feeling positive emotions, positive relations with others, spiritual growth, having a sense of meaning and purpose in life as well as a greater sense of empowerment. Each of these categories is presented in Table 2.

**Table 2:** Perceived effects of unforgiveness on mental wellbeing

Negative effect on mental health/affect
Felt emotionally disturbed, depression, stress
No sense of peace, fear, guilty, lack confidence
Depletes energy, Felt static, worse, bitter, worthless
Blood rushing through body
Rage, darkness
Negative effect on mental health/cognitive
Inability to think clearly, suicidal thoughts
Thoughts of harming others
Barriers to growth
Stops you from moving on, freezes mind, less dynamic
Stuck in a rut
Lack of meaning and purpose in life
Unable to form new relationships
Transferring anger/bitterness into new relationships
Constant falling out

### Reductions in Negative Affect

All participants spoke about experiencing reductions in event-specific negative emotions such as anger, hatred, animosity and rage. For instance, one participant stated the following:

I no longer have any animosity towards him (father). If he said something that would annoy me before or have feelings of animosity, without having to think about it, it just drops off me. I don’t have any negative mental reaction to him. I just have an accepting and compassionate view of him. I no longer say to myself, quietly in my mind, “I really hate you” (Alan, Theosophist)

Another Theosophist also indicated how feelings of unforgiveness were causing him to feel generally depressed. However, forgiveness enabled him to overcome his mood disorder and feelings of bitterness and instead ‘heal’ his mental health (Lyndon, Theosophist). Similarly, an ACIM participant indicated that although he was experiencing suicidal thoughts and a lot of ‘darkness’, forgiveness enabled him to let go of wanting to harm himself, change his depressive state and experience greater happiness (Quinn, ACIM) (Table 3).

**Table 3:** Effects of state forgiveness on mental wellbeing

Reduction in negative affect
Anger, hatred, rid of burden, animosity, bitterness
Irritation, depression, conflict
Positive affect
Peace, content, joy, love, felt better, calmer, freedom from fear, happy, uplifted, inspired, compassion, positive, felt normal, confidence, vitality, autonomy
Positive relationships
Accepting other (positive attitude; accepting good and bad qualities), loving other, caring for/helping other, understanding, closer, value people, tolerant, less breakups, meaningful relationship, reconciliation, less reactive, more open, pleasurable, healthier for kids, moving forward, healed relationships
Personal growth
Spiritual transformation, meaning and purpose to life
Sense of empowerment
Stronger, independent, confident, hopeful, calmer, in control

### Positive Affect

For all religious/spiritual participants, forgiveness appeared to be related to an improvement in general positive emotions and not just event-specific feelings such as love and compassion. Some examples of common descriptions by participants regarding how they felt after practicing forgiveness included (feeling) ‘content’, ‘joy’ (Brandon, ACIM), ‘calm’, ‘better’ (Trisha, (ACIM), ‘happier (Quinn, ACIM), ‘uplifted’ (Stuart, ACIM), ‘stronger’ and ‘independent’ (Amira, Muslim). Feelings of inner peace were also commonly described by Muslim and new religious participants. Reflecting the views of other ACIM and Muslim participants, one respondent who was able to overcome the depression he was feeling from unforgiveness stated the following regarding the outcomes of his forgiveness practice:

Going through the last few months, although there was conflict, there was rage, there was everything, I’m just much happier, more peaceful within myself; I don’t take things as seriously as I used to. (Quinn, ACIM)

Another ACIM participant described how he experienced a reduction in negative affect followed by an increase in positive emotions as a result of forgiving:

I ended up feeling like I was in a different state of mind that I usually am in, much more positive and loving. And there was also the negative in the sense of negating that feeling of being stuck in the anger. I didn’t feel stuck in the anger anymore. (Stuart, ACIM)

### Positive Relationships

The majority of participants described experiencing improved relationships such as being able to work together constructively, experience intimacy with their partners, empathy, open-mindedness and acceptance as a result of their practice of forgiveness. For example, common themes reported by ACIM participants included: being able ‘to love (in an) environment that had been so hostile’, ‘willing(ness) to work together’ (Quinn, ACIM), ‘closer family relationships’, ‘understanding and love for each other’ (Bill, ACIM), ‘more open’, ‘tolerant’ (Trisha, ACIM) and people ‘more valuable, real, worth loving’ (Stuart, ACIM). ACIM participants reported that relationships were a significant component of their wellbeing; as one participant stated ‘there can be no wellbeing unless your relationships are healed’ (Brandon, ACIM), and forgiveness was a key strategy utilised for this purpose:

It (forgiveness) has totally healed my relationship with my parents. We get along all the time now. There’s less in the way of getting on and they can see it in you that you look at them in a nicer, more loving way. (Brandon, ACIM)

One Muslim participant described how her relationship with her husband had improved. She stated that ‘we can talk to each other about anything and everything’, ‘we trust each other’ and that their improved relationship made it ‘healthier for the kids’, (Amira, Muslim). The Buddhist participant, Jax, felt that his relationship with his father was more pleasant and that he had developed compassion and understanding towards him, whilst Alfred, the secular/atheist participant, was able to maintain a friendly relationship with his former partner who had rejected and offended him. One of the Theosophist participants also stated that he ‘gradually thought of (his father) in a less negative light’ and had developed ‘an accepting and compassionate view of him’ (Alan, Theosophist). Another Theosophist participant felt ‘compassion, kindness and peace’ towards an ex-partner who had severely deceived him and also found that his general relationships improved:

You develop compassion, kindness and peace through forgiveness that leads you to healthier relationships with people. (Lyndon, Theosophy)

### Spiritual Growth

Most participants spoke of experiencing spiritual development. For new religious participants in particular, this took the form of spiritual growth whereby they experienced a shift in understanding about their own self, which also appeared to give them a sense of meaning and purpose to life. For instance, one participant (Lyndon, Theosophist) spoke of how he ‘learned something’ about human nature and the importance of insight. He stated that through a lack of forgiveness he was identifying with a superficial self, which was selfish and lacked concern for others, and that unforgiveness resulted in a state that caused him to feel depressed and was a barrier to his spiritual and social wellbeing. In contrast, forgiveness enabled him to manifest beliefs about his ‘true self’, which he described as inherently selfless.

Participants felt that their understanding of life and of people expanded as they developed a new sense of (spiritual) self, in which they placed more emphasis on concern for the welfare of others, as well as being compassionate towards themselves and others, which were key elements of their spiritual development:

I felt it was a real turning point for me (forgiving); it was really occasioned by a sense of tremendous leap forward in terms of my own spiritual journey. My goal is spiritual awakening and I think forgiveness is the key in that process. (Stuart, ACIM)

### Sense of Empowerment

Forgiveness also appeared to give participants a sense of empowerment and control as they were able to overcome negative feelings associated with unforgiveness such as hatred and the desire for revenge, and change the state they were in for the better. For instance, common themes found among religious/spiritual participants were feeling ‘stronger’, ’independent’ (Amira, Muslim), ‘confident’, ’hopeful’, ’calmer’ (Trisha, ACIM) and ‘in control’, (Jax, Buddhist). Many ACIM participants stated that by ‘healing’ the mind (through forgiveness), they had improved their health and wellbeing (Quinn, ACIM). The Buddhist and new religious participants in particular believed that they could choose whether or not to prolong the initial suffering experienced. This may have been related to their beliefs about taking responsibility for how they reacted and what they focused upon. Brandon (ACIM) suggested that any feelings of resentment were caused as a result of his own thought processes, which he believed he could change at any point, and stated that accepting responsibility was ‘a lot more empowering’.

For these participants, the choice appeared to be between identification with ego (i.e. fear, unforgiveness) or with the ‘higher self’ (i.e. love/compassion) and by choosing to forgive they appeared to be able to reverse the effects of the harm through inner (cognitive, emotional) change. Letting go, or forgiveness, as one Buddhist stated, was a means of taking control of his life:

If I focus on what the other person is doing then all the power is in their hands; they have complete control over me. Letting go is a way of taking back control. (Jax, Buddhist)

### Factors Influencing Forgiveness and Wellbeing

Participants also highlighted a number of factors that seemed to influence their forgiveness and wellbeing levels. For example, many stated that blaming, lack of acknowledgment, powerlessness, ruminating, revenge, ongoing transgressions and physical proximity obstructed their forgiveness process. Of the participants that described barriers to forgiveness, the same respondents also spoke about factors that promoted their forgiveness practice. Key themes assisting forgiveness among respondents were: a sense of connectedness with others, focusing on positive qualities, accepting responsibility, acceptance of karma, beliefs about being of benefit to others, meditation, self-observation, prayer, empathy, persistent effort (by the offender) to repair the situation as well as talking to and support from friends.

For the majority of religious/spiritual participants (namely ACIM, Muslim, Buddhist and Theosophist), forgiveness appeared to have ties with wider dimensions of wellbeing. For instance, they described experiencing spiritual development, positive relationships, positive affect, and a sense of meaning and purpose. On the whole, however, it seems that participants, who largely applied inner transforming strategies, without necessarily expecting a change in external conditions, appeared to experience greater levels of forgiveness and mental wellbeing. Unforgiveness on the other hand, which appeared to be reinforced by factors such as blame, feeling powerless, ruminating and desires for revenge, had a variety of negative effects on participants mental, emotional and social wellbeing. Whilst the themes described here are tentative, there is indication that regular practice of a combination of inner transforming factors such as meditation, responsibility and helping others/connectedness may be effective in facilitating forgiveness of a specific transgression among certain religious/spiritual samples.

## Discussion

The central purpose of this study was to explore connections between state forgiveness and a variety of psychological wellbeing outcomes and to investigate what factors may influence a forgiveness–wellbeing link. Analysis of the qualitative interviews revealed relationships between unforgiveness and depression (Maltby et al. 2005). Much of the previous research literature focuses primarily on associations between trait and state forgiveness and common mental health disorders such as depression and anxiety (Toussant and Webb 2005). However, in the present study participants also provided insights in terms of a variety of other problems that appeared to result from unforgiveness. For example, feelings of guilt, worthlessness, a lack of energy (i.e. depleted, static, slow), lack of confidence and fear. Participants also indicated how unforgiveness negatively affected their cognitive abilities because they were unable to think clearly, had suicidal thoughts, as well as thoughts of wanting to murder. A majority of participants felt unforgiveness to be a barrier to growth as they were unable to move forward in life and felt ‘stuck in a rut’. In particular, many stated that feelings of anger and bitterness were transferred into new relationships, which resulted in arguments and constant falling out. Other dimensions of wellbeing that appeared to be affected were feelings of a lack of meaning and purpose in life. Thus, the exploration of participant’s experiences of unforgiveness highlighted the prevalence of a wider range of mental health problems but also identified the negative effect unforgiveness had on other aspects of general functioning, an area that has to a large extent been unexplored.

Previous experimental research has also shown that forgiveness of real-life offences can facilitate the development of general positive affect such as feelings of hope, gratitude and happiness (Rye et al. 2012; Freedman and Enright 1996; Allemand et al. 2013). Similar results were also observed in this study, which also revealed a more specific and wider set of positive affective outcomes that resulted from forgiving such as feelings of peace, contentment, joy, calmness, freedom, confidence, vitality and autonomy.

In addition to increasing positive affect, another factor that emerged from participant’s descriptions of practicing forgiveness was spiritual growth. This encompassed an understanding of a spiritual self that was more connected with others and entailed a process of learning and understanding about human relationships, both of which gave a new sense of meaning and purpose to participant’s lives. These findings are consistent with experimental research conducted by Luskin et al. (2005) in which he reported that forgiveness treatment resulted in increased levels of spiritual wellbeing such as personal growth/compassion and embracing life’s fullness.

Another unique theme that emerged from participant’s accounts was that of feeling empowered, with participants stating that they felt stronger, independent, confident, calmer and more in control as a result of being able to change their negative psychological state to a positive one. No known studies have specifically explored this category, but prior research (Allemand et al. 2013) has indicated links between forgiveness and confidence. It is important to note, however, that empowerment, such as feeling a sense of control, confidence, ability to change a negative situation to a positive one, may be both a facilitator and outcome. For instance, research conducted by Hill and Allemand (2010) suggested that environmental mastery assisted trait forgiveness, whilst this study indicates that powerlessness inhibited forgiveness. Therefore, the causal direction may run both ways.

There have been some contradictory findings regarding the effects of forgiveness on interpersonal relationships. In line with the findings of this study, some have found that forgiveness improves marital satisfaction (Baskin and Rhody 2011) and increases positive relations with others (Ostendorf et al. 2011). However, others have found that state forgiveness does not improve relationships with others (Ripley and Worthington 2002). All of these previously cited studies have, however, assessed event-specific relationships; that is, effects of forgiveness between couples taking part in the research or between participants and those who hurt them. No known experimental studies have assessed the effects of forgiveness programs on general relationships. Whilst in the majority of cases the qualitative study participants stated that it was their relationship with the specific offender that improved, they also alluded to experiencing benefits beyond this. For example, participants said that forgiveness enabled them to ‘move forward’, and to ‘develop healthier relationships with (their) children’ which felt ‘more open’. They also stated that they were able to let go of a variety of negative thoughts and emotions such as bitterness, anger, stress and not being able to think clearly, factors which were hindering them from developing new relationships. Thus, the interview findings suggest that forgiveness positively influenced a number of different aspects of individuals general functioning.

Previous longitudinal research conducted by McNulty (2011) has also found that a greater tendency to forgive is associated with increased psychological and physical aggression over a 4-year period whilst those who had a lower propensity to forgive experienced reductions in psychological and physical aggression over time. However, results from the present study indicated that participants experienced more positive relationships with the offender and there was no suggestion of an increase or in fact any further violence over time. In the case of the two participants who experienced domestic violence, Amira said she was much happier in her marriage whilst Rana chose to divorce her husband but was still able to forgive him and noticed that he behaved more positively towards her and the children. Respondent five, Jax, also stated that he experienced ongoing transgressions by his father and subsequently limited the time he spent with him. However, he continually applied forgiveness in response to being hurt and stated that their relationship was consequently more positive. Nevertheless, further research that specifically examines the reduction or increase in offences post (state) forgiveness within the context of ongoing close relations is warranted.

This study has further contributed to knowledge by highlighting factors that may influence a forgiveness–wellbeing link. Most previous research has focused on the role of religious and secular factors in influencing dispositional forgiveness, and very little is known about novel mechanisms employed by religious and secular people that may assist or restrict forgiveness of real-life interpersonal offences. The present study extends on previous research by highlighting potential effect modifiers among under-studied samples. The findings suggest that specific beliefs and practices, including viewing an offender as spiritually similar or different, responsibility/karma, blaming, restitution/apology, as well as practices such as meditation and prayer, may help to facilitate or act as barriers to forgiveness. The study also highlighted that those who more strongly emphasised practicing unconditional forgiveness appeared to experience greater *levels* of state forgiveness and subsequently wellbeing than participants wanting the offender to make amends (Muslim; secular/atheist). In the present study, most participants appeared to benefit from different processes of forgiveness, albeit to different degrees. However, prior research has indicated that conditional forgiveness is associated with greater psychological distress and reduced levels of psychological wellbeing among a sample of older Christian adults (Krause and Ellison 2003).

### Implications

The present study indicates that beyond reducing depression and anxiety, state forgiveness may be a key facilitator of general cognitive, emotional and social wellbeing outcomes. Future studies should as such include a wider range of psychological health measures so that greater awareness can be raised regarding the costs and benefits of forgiving or unforgiveness. Also, consistent with prior research (Kidwell et al. 2012), this study has highlighted important differences in how participants forgave and the perceived effect this had on their psychological health; the findings suggest that in working towards assisting clients to overcome a range of interpersonal hurts, therapists may need to draw upon specific beliefs and practices of importance to individual clients in order to help them to forgive.

### Limitations and Future Directions

Whilst the qualitative component of this study has provided an in-depth understanding of the experiences of forgiveness among diverse and under-studied groups, it has a number of limitations. For instance, the number of Buddhist, Secular/Atheist, Theosophist and Muslim participants who took part in this study was quite low, and therefore, the findings should not be generalised to others affiliated with these groups. Moreover, whilst the study recruited a demographically diverse group of participants, further studies that include more participants of different genders, ethnicities and socio-economic backgrounds as well as an assessment of their personality characteristics are needed to assess participant’s perceptions about the effects of these factors on their forgiveness process. Further research is also needed to examine the influence of factors that may modify the effect identified in the present study among diverse groups and to investigate whether forgiving real-life interpersonal offences is causally related to general wellbeing outcomes.

## References

[CR1] Allemand M, Hill LP, Ghaemmaghami P, Martin M (2012). Forgivingness and subjective well-being in adulthood: The moderating role of future time perspective. Journal of Research in Personality.

[CR2] Allemand M, Steiner M, Hill LP (2013). Effects of a forgiveness intervention for older adults. Journal of Counselling Psychology.

[CR3] Baskin TW, Enright RD (2004). Intervention studies on forgiveness: A meta-analysis. Journal of Counseling & Development.

[CR4] Baskin TW, Rhody M (2011). Supporting special needs adoptive couples: Assessing an intervention to enhance forgiveness, increase marital satisfaction, and prevent depression. The Counseling Psychologist.

[CR5] Chan WD (2013). Subjective well-being of Hong Kong Chinese teachers: The contribution of gratitude, forgiveness, and the orientations to happiness. Teaching and Teacher Education.

[CR6] Chida Y, Steptoe A (2009). The association of anger and hostility with future coronary heart disease: A meta-analytic review of prospective evidence. Journal of the American College of Cardiology.

[CR7] Department of Health. (2011). *No health without mental health: A cross*-*government mental health outcomes strategy for people of all ages*. Accessed May 24, 2015.

[CR8] DiBlasio FA (1998). The use of a decision-based forgiveness intervention within intergenerational family therapy. Journal of family Therapy.

[CR9] Enright RD, Fitzgibbons RP (2000). Helping clients forgive: An empirical guide for resolving anger and restoring hope.

[CR10] Freedman SR, Enright RD (1996). Forgiveness as an intervention goal with incest survivors. Journal of Consulting and Clinical Psychology.

[CR11] Goldman BD, Wade N (2012). Comparison of forgiveness and anger-reduction group treatments: A randomized controlled trial. Psychotherapy Research.

[CR12] Hill PL, Allemand M (2010). Forgivingness and adult patterns of individual differences in environmental mastery and personal growth. Journal of Research in Personality.

[CR13] Huppert FA (2009). Psychological well-being: Evidence regarding its causes and consequences. Applied Psychology: Health and Well-being.

[CR14] Kidwell EJ, Wade N, Blaedel E (2012). Understanding forgiveness in the lives of religious people: The role of sacred and secular elements. Mental Health, Religion & Culture.

[CR15] Krause N, Ellison GC (2003). Forgiveness by God, forgiveness of others, and psychological well-being in late life. Journal for the Scientific Study of Religion.

[CR16] Krug EG, Dahlberg LL, Mercy JA, Zwi ZA, Lozano R (2002). World report on violence and health.

[CR17] Lundahl WB, Taylor JM, Stevenson R, Daniel KR (2008). Process-based forgiveness interventions: A meta-analytic review. Research on Social Work Practice.

[CR18] Luskin FM, Ginzburg K, Thoresen CE (2005). The efficacy of forgiveness intervention in college age adults: Randomized controlled study. Humboldt Journal of Social Relations.

[CR19] Macaskill A (2005). The treatment of forgiveness in counselling and therapy. Counselling Psychology Review.

[CR20] Maltby J, Day L, Barber L (2005). Forgiveness and happiness. The differing contexts of forgiveness using the distinction between hedonic and eudaimonic happiness. Journal of Happiness Studies.

[CR21] McNulty JK (2011). The dark side of forgiveness: The tendency to forgive predicts continued psychological and physical aggression in marriage. Personality and Social Psychology Bulletin.

[CR22] Osterndorf C, Enright R, Holter A, Klatt J (2011). Treating adult children of alcoholics through forgiveness therapy. Alcoholism Treatment Quarterly.

[CR23] Park J, Enright RD, Essex MJ, Zahn-Waxler C, Klatt JS (2013). Forgiveness intervention for female South Korean adolescent aggressive victims. Journal of Applied Developmental Psychology.

[CR24] Ripley JS, Worthington EL (2002). Hope-focused and forgiveness-based group interventions to promote marital enrichment. Journal of Counseling and Development.

[CR25] Royal College of Psychiatrists. (2010). *No health without public mental health: The case for action*. http://www.rcpsych.ac.uk/pdf/Position%20Statement%204%20website.pdf. Accessed July 2014.

[CR26] Rye SR, Fleri MA, Moore DC, Worthington LE, Wade GN, Sandage JS, Cook MK (2012). Evaluation of an intervention designed to help divorced parents forgive their ex-spouse. Journal of Divorce & Marriage.

[CR27] Sastre MTM, Vevinsonneau G, Neto F, Girard M, Mullet E (2003). Forgivingness and satisfaction with life. Journal of Happiness Studies.

[CR28] Shectman Z, Wade N, Khoury A (2009). Effectiveness of a forgiveness program for Arab Israeli adolescents in Israel: An empirical trial. Peace and Conflict.

[CR29] Toussaint L, Friedman P (2009). Forgiveness, gratitude, and well-being: The mediating role of affect and beliefs. Journal of Happiness Studies.

[CR30] Toussaint L, Webb JR, Worthington EL (2005). Theoretical and empirical connections between forgiveness, mental health and wellbeing. Handbook of forgiveness.

[CR31] Wade NG, Hoyt WT, Kidwell EM, Worthington EL (2013). Efficacy of Psychotherapeutic Interventions to Promote Forgiveness: A Meta-Analysis. Journal of Consulting and Clinical Psychology.

[CR32] Wade GN, Worthington LE (2005). In search of common core: A content analysis of interventions to promote forgiveness. Psychotherapy: Theory, Research, Practice Training.

